# People overlook subtractive changes differently depending on age, culture, and task

**DOI:** 10.1038/s41598-024-51549-y

**Published:** 2024-01-11

**Authors:** Joshua Juvrud, Laurence Myers, Pär Nyström

**Affiliations:** 1 Department of Game Design, Uppsala University, Campus Gotland, Visby, Sweden; 2 Brown University, Providence, USA; 3 Present Address: Department of Computer Science, University College London, London, UK; 4 Department of Psychology, Uppsala University, Uppsala, Sweden

**Keywords:** Psychology, Human behaviour

## Abstract

Previous work has explored transformative strategies that adds or removes components to change an original structure or state, and showed that adults tend to search for additive solutions far more often than subtractive ones. In the current study, we replicated a Lego building task and a grid-based symmetry task from a previous study, and also introduced a novel digital puzzle task. We investigated limitations in the previous study as well as extended the investigation of the subtraction neglect in a sample of children and across two cultures. Results partially confirm previous results, and extends the literature by showing that 9–10 year old children were more likely to ignore subtractive transformations than adults. However, we found both task-based and cultural variations in strategy use in adults from Sweden and the USA. We conclude that a subtraction neglect involves complex cognitive processes that are dependent on the task, culture, and age.

## Introduction

Solving a problem to achieve a given goal requires the use of strategies to evaluate potential actions and their end states^1^. Strategies are selective, goal-directed, and intentional to determine whether any given action produces an improved state^2^. In order to evaluate possible actions, mental models are used to determine possible transformations from the original state into a novel state, often based on prior knowledge and environmental cues. Prior work across a variety of cognitive domains and operations^3–5^ has identified two critical transformative strategies, subtraction and addition, which serve as action categories that remove components from or add components to the original structure or state. For example, a subtractive strategy would remove a number of blocks from a structure, or make cuts to a financial budget, in order to achieve an intentional end goal-state. The key is that individuals may add or subtract to fulfill any number and types of goals.

The problems that people face on a day-to-day basis can often be solved using either an additive or subtractive strategy or a combination of both. For example, in a recent paper Adams et al.^3^ asked participants to stabilize a Lego structure that was off balance by adding new blocks to reinforce a single corner block, or the corner block could just as reasonably be subtracted from the structure to reach the same stabilized goal, resulting in the corner block flush on the layer below. The authors conducted an extensive study using eight separate tasks and concluded that adults showed a distinct subtraction neglect in transformative solutions, even when the subtractive solution is more efficient, and even when individuals were verbally primed with the subtractive solution. If generalizable, this is a remarkable finding with implications for most forms of human decision-making and therefore merits some scrutinization and at least some replication attempts.

Other work has also shown a subtraction neglect in cognitive processes^6–8^, but there remain important considerations in previous findings that have yet to be examined. For example, Adams et al.^3^ note that certain heuristics and cues may have an impact on the condition in which advantageous subtractive solutions are used. For instance, explicit instructions to “reduce” costs (or priming subtractive solutions) would favor subtractive solutions. Nonetheless, even with subtractive cues, adult participants still favored the additive solutions across all of their tasks. They, therefore, concluded that adults tend to default to additive strategies and ignore subtractive ones.

The findings suggesting a subtraction neglect that may generalize across cognitive processes merit conceptual extensions. Indeed, other studies have raised concerns, such as potential methodological limitations in Adams et al.’s experiments. For example, in the Lego task, instructions to “add or subtract” were not counterbalanced, which could lead to a bias simply because all participants were first primed with “add.” Additionally, the orientation of the Lego tower and the placement of the figure could have drawn participants’ attention away from the single supporting block, reducing the likelihood of them recognizing the option of removing it^9^. Similar conceptual work has examined tower building tasks not unlike the Lego task^7^ (Varallyay et al. 2023), however, these tasks relied on social learning to solve a novel problem.

For our part, we found alternative explanations for the grid symmetry task conducted by Adams et al.^3^. In particular, this task may be influenced by Gestalt psychology, which involves the organization of stimuli into wholes rather than parts. One principle of Gestalt psychology, the simplicity principle, is organizing stimuli into the most simple and all-encompassing structure, such as a cohesive shape, even if the structure is incomplete. This might explain participants’ bias toward adding to complete the whole of a perceived symmetrical shape (such as a square; Ref.^10^). In the case of the grid symmetry task, participants might have perceived the green square in the upper left quadrant as part of a whole, leading them to add three more sets of squares to complete the whole (see Fig. S2).

Finally, it may be that priming through social learning, including explicit verbal instruction and learning through observation, may not have the same effect as cueing behavior through a reinforcement of successful solutions through generated own-experience behavior^11,12^. For example, learning through trial-and-error and repeated attempts that a solution can be solved through addition or subtraction might be more likely to influence subsequent behavior than verbal instruction alone.

Remaining questions raised by Adams et al.^3^ in the discussion of their results allude to the origins of a subtraction neglect bias. It is still unclear to what extent such a bias is acquired under the influence of sociocultural environments, or at what age such a bias might be learned. From an evolutionary perspective, Adams et al. suggested that humans defaulting to additive solutions might be more often beneficial overall. Indeed, there are many instances in which such heuristics are beneficial to survival (e.g., reproduction). Some prior work has shown in children as young as 4 years old a tendency to additive solutions in both language and object-based tasks^6,7^. The developmental trajectory of such a subtraction neglect is still unclear, particularly in tasks that do not rely on demonstration. It is possible that this observed subtraction neglect becomes stronger across early development and into adulthood, perhaps strengthened through social and cultural factors. For instance, prior research shows that in certain problem-solving tasks, children become significantly more efficient in their choice of a solution strategy: that is, they increasingly choose solution strategies requiring fewer physical actions to reach a goal^2^.

It is here worth considering modern trends in Western society that tend to overlook subtraction solutions, suggesting potential social and cultural explanatory factors. For example, modern tendencies to have overburdened minds and schedules^13^ and modern American ideologies of “more is better”^14^. Such considerations speak to possible cultural effects, which were presented by Adams et al.^3^ as a possible explanation, whose findings were only based on samples collected in the United States. Such cultural factors could differentially strengthen or weaken a subtraction neglect tendency across development, leading to more or less of a subtraction neglect being present in adults.

Based on the presented introduction, the current paper had three aims: (AIM1) to replicate the findings of Adams et al. in a new sample of adults using two similar tasks, the Lego and grid symmetry task, while addressing methodological concerns related to these tasks; (AIM2) to investigate the effect of experience by varying culture (American/Swedish) and age (9–10 year old children), and (AIM3) to extend the findings of a possible subtraction neglect to a novel task, called the Ludum game, where priming occurs through generated own-experience, rather than explicit instruction or observation.

## Method

### Participants

The total number of participants tested included 58 adults living in either Sweden or the United States (Swedish adults n = 27, American adults n = 31; M age = 21.18 years, SD = 1.89), and 58 children aged 9–10 living in Sweden (M age = 10.10 years, SD = 0.21). See Table 1 for a breakdown of the sample size for each individual task. All adult participants provided written informed consent to participate in the study. Both parents/legal guardians provided written informed consent for their child’s participation. The methods and protocol of the study were conducted in accordance with the standards specified in the 1964 Declaration of Helsinki and were approved by the local ethics committee of Uppsala University, The Swedish Ethical Review Authority (2022-03420-02).

**Table 1:** The number of participants included for each task.

	Adults		9–10 year olds
	Swedish	American	Swedish
Lego task	*n* = 25	*n* = 30	*n* = 22
Symmetry task	*n* = 25	*n* = 30	*n* = 46
Ludum task	*n* = 27	*n* = 28	*n* = 43

### Lego task

We adapted the Lego task from Adams et al.^3^ experiment 1 to additionally examine alternative explanations for performance. Participants were given a cube-shaped Lego structure with a platform on the top (Fig. S1) and a cup of extra Lego bricks. The platform was supported by one small brick on the corner on top of the structure. Researchers instructed participants to stabilize the platform such that a toy car can be placed on top of it, without falling on the monkey figure standing underneath. The instructions followed a script, “You may add or take away (take away or add) Lego bricks however you like, but the structure and platform must be higher than our Lego buddy, so that they can stand underneath the platform. Try to solve the task using as few bricks as possible.” To address possible priming effects^9^, we counterbalanced the instructions as “add or take away” or “take away or add,” as well as the orientation of the Lego structure to account for possible cues for additional strategies (four counterbalance conditions).

### Grid symmetry task

We adapted the grid symmetry task from Adams et al.^3^, while also extending their findings to take into account additional configurations, including gestalt principles. In the original task, only four configurations were used, which may have limited the generalizability of the original findings, and we therefore added two more levels with gestalt laws suggesting addition, and two levels which suggested subtraction. We first provided instructions including the goal of making the quadrants symmetrical. The demonstration was counterbalanced: participants were told the task could be completed by “Tapping on an empty square to add a green tile, or by tapping a green tile to remove it” or “Tapping a green tile to remove it, or by tapping on an empty square to add a green tile.” As the researcher described adding or removing a green tile, they also demonstrated on the tablet.

Stimuli for a symmetrical pattern task were displayed on a Lenovo tablet with dimensions of 247 mm × 171 mm × 9.6 mm using Unity Real-Time Development Platform engine and software. Four quadrants were shown on the screen with arrangements of green tiles. Participants were asked to make all four quadrants symmetrical about the center by tapping on empty squares to add tiles or by tapping on existing tiles to remove them. They were asked to use the fewest moves possible to complete the tasks. Once a symmetrical solution was met, the participant could move forward with the next level. The first screen acted as a practice round for the experimenter to demonstrate how the task worked. There were then eight levels for participants to work through. Patterns in each level either followed the Gestalt principles and required adding or removing tiles to yield a complete shape, or did not follow Gestalt principles. The most efficient solutions (i.e., with the fewest moves) for each level differed in the number of additive and subtractive moves (Fig. S2).

### The Ludum task

The purpose of this task was to extend investigations of subtraction neglect to a novel task that encouraged learning through self-experience and explorative play. To minimize the potential effect of social learning, verbal and written instructions focused on overall usability (i.e. controlling the game) without details about specific game mechanics or possible logical operations needed to complete the task.

Participants played a digital game called Ludum on a computer. The game was created and run using the Unity Real-Time Development Platform engine and software. The participant entered a series of rooms and interacted with objects in the rooms to unlock the door to the next room. They could move around using the WASD keys and interact with objects using the left mouse button.

After starting the game, researchers provided a brief description of the controls and goal of the task, i.e., to move to the next room. Participants began the game in a practice room with no objects, and detailed written instructions of the controls were provided within the task, including how to move and orient the camera, interact with objects, and how to open and walk through the door. Researchers aided and answered questions about the controls only in the practice room.

After the practice room, participants entered a sequence of rooms with blocks of different shapes and colors, a detector platform, a red button, and a locked door. The door would only open when the participant pressed the red button while the correct combination of blocks had been placed on the detector.

There were two versions of the task: an addition cue condition and a subtraction cue condition. The two conditions differed in the training rooms and the learning phase of the task. In the training phase, participants in the addition cue condition first entered the addition rooms (Fig. S5), where putting all blocks on the detector unlocks the door. In the subtractive cue condition, participants had to instead remove all blocks away from the detector to unlock the door. In the learning phase, participants had to learn which particular block opened the door. In additive rooms, a particular block had to be put on the detector, while a block had to be removed from the detector in the subtractive rooms. These rooms repeated a total of three times, with the color and shape of the blocks counterbalanced across participants and conditions.

Finally, participants entered the test room. In the test room, there are three possible ways to open the door (see Fig. 3): (1) add a block onto the detector (additive strategy), (2) remove a block from the detector (subtractive strategy), (3) both remove a block from the detector and add a block onto the detector (both subtractive and additive strategy. See Fig. S6. In the test room, equal blocks were placed both on and off the detector, and thus requiring a choice of whether to add blocks, subtract blocks, or a combination of both. We determine efficiency as the number of actions required to activate the door, that is, the number of blocks added or removed. Combining both adding and subtracting blocks was therefore less efficient, because it required more actions of picking up, moving, and placing multiple blocks.

## Results and discussion

Overall, our results suggest that subtraction neglect is highly task-, culture-, and age-dependent, and may not be as generalizable as suggested by Adams et al.^3^. Statistical analyses and the generation of figures and plots were carried out using IBM SPSS Statistics software and R.

### AIM1

**Figure 1:**
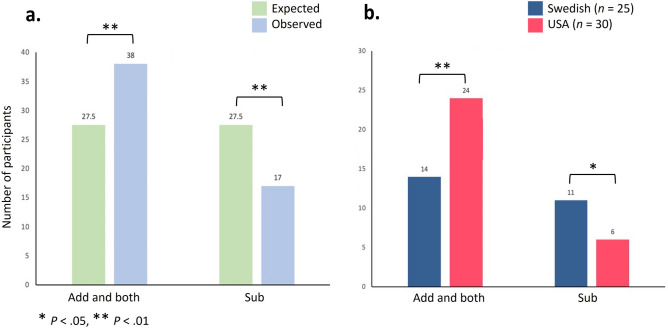
Lego task: (**a**) Observed and expected strategy distribution among all adults when looking at subtractions against the other two less efficient (add, both) strategies. (**b**) Adult Americans (n = 30) accounted for most of the participants in the add or both category, while Swedish adults (n = 25) accounted for most of those in the subtract category.

In contrast to Adams et al.^3^, we found mixed results in both replicated tasks (Lego and grid symmetry tasks,see Table 1 for an overview of the tasks). The grid symmetry task, with only neutral stimuli and adult participants, did not show any difference between addition and subtraction, *t*(52) = -0.453, *p* = 0.653. In the Lego task, when looking at the data from all of the participants, we found no bias towards subtraction neglect or a combination of both strategies. These findings are not consistent with the ones found by Adams et al.^3^. However, when combining additive strategies with strategies that used both addition and subtraction, there was a clear bias for this category compared to strictly subtractive solutions (see Fig. 1a and b). In the Lego task, counterbalancing verbal cues in the instructions did not influence participants’ transformative solutions. The order in which “adding bricks” and “taking bricks away” were mentioned in instructions for the Lego task and positioning of the stimuli did not make participants more or less likely to adopt certain strategies.

### AIM2

**Figure 2:**
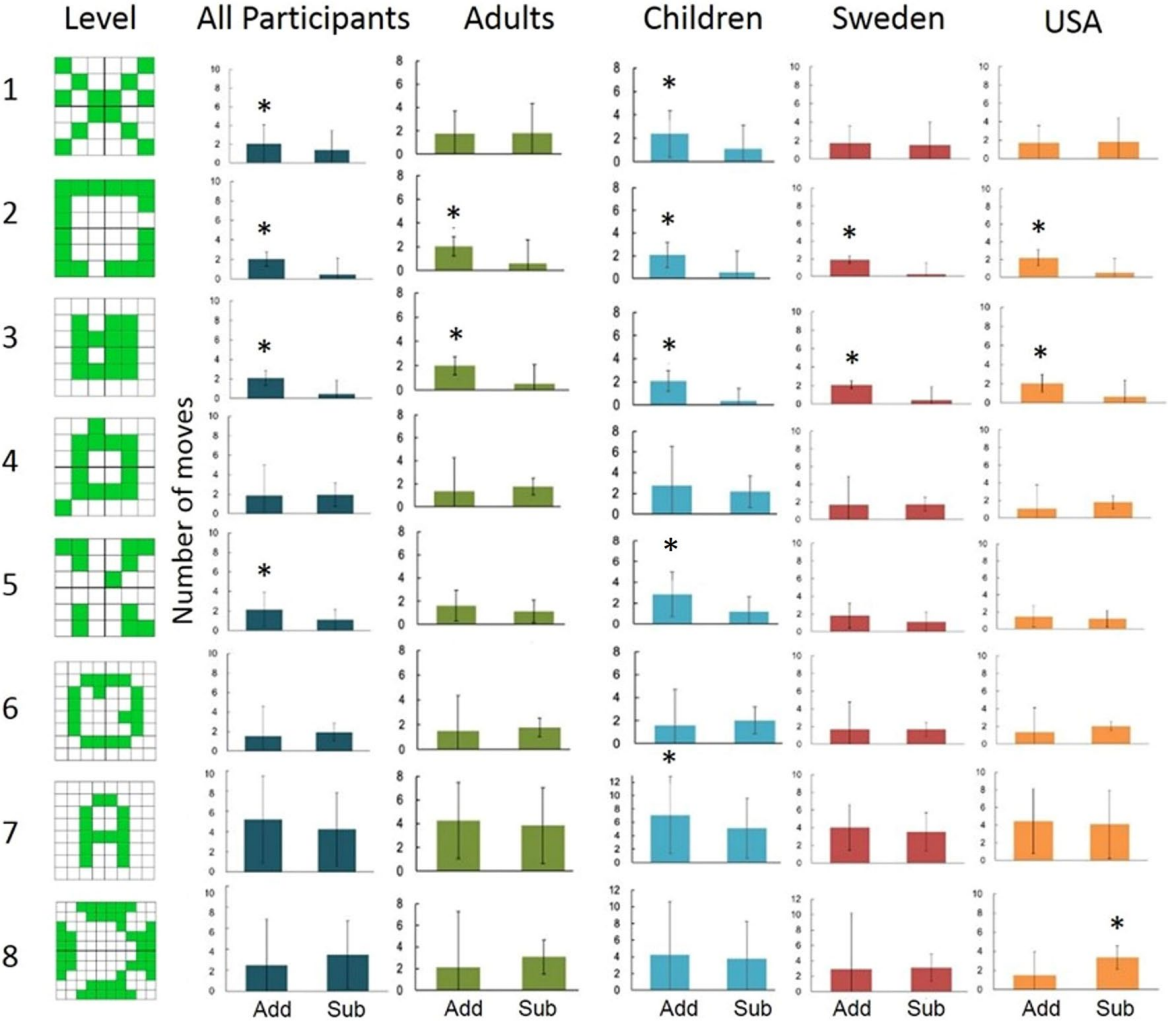
Grid symmetry task: The number of adding/subtracting moves on all grid symmetry task levels for all participants, adults, children, USA adult participants, and Swedish adult participants. * indicates p < 0.05. Error bars represent standard deviation.

We examined performance across all three tasks in both Swedish and American participants. In the grid symmetry task, we examined more grid configurations than Adams et al.^3^. Some of the patterns (levels) were designed to promote Gestalt principle goals: the most efficient solution was to add for some levels and to subtract for others. We performed separate repeated measures ANOVAs for age and nationality, which showed significant main effects for the repeated gestalt law factor (neutral / addition / subtraction), both for age (*F*(2, 136) = 7.725, *p* < 0.001, η^2^ = 0.053) and nationality (*F*(1.721, 87.748) = 15.469, *p* < 0.001, η^2^ = 0.113), but no other main or interaction effects. However, planned post-hoc tests found several cultural differences in the grid symmetry task when level-wise comparisons were performed (Fig. 2). Regarding cultural differences between American and Swedish adults in the transformative solutions that were used, Swedish adults were more likely to utilize subtractive solutions. There was some variation between people living in the US and Sweden in the Lego task and the grid symmetry task, although not consistently across all configurations. While Sweden and the United States have many cultural differences that could explain these findings, such as a decrease in the value placed on individual success in Sweden^15^, both share many Western ideals such as a strong sense of individualism^16^. Studies have suggested that additive learning is intuitive^8^, and that particularly in the West, children and adults may spontaneously solve problems (by combining possible elements) in a goal-directed, problem-solving context. It will be important to examine additional cultural contexts that are further separated from common Western ideologies, to show the possible range of cultural impact on addition and subtraction strategy use.

Regarding age differences, we found that children aged 9–10 years were more likely to use additive strategies than adults in the grid symmetry task (Fig. 2). A possible interpretation for these findings could be that children at this age have little incentive to seek the most efficient solution, and instead choose what is safest (or the solution that maximizes the likelihood of success). This is not consistent with certain developmental theories of efficient actions, such as the teleological stance^17,18^,however, the children included in the current study are at an age where social learning could have an influence on their transformative solutions. These findings, coupled with developmental data in similar tasks^6,7^, speak to a possible universal trend toward addition and away from subtraction and may point toward this heuristic having a developmental trajectory. Further research should examine children longitudinally across development, particularly beginning at younger ages, to further understand the developmental trajectory of subtraction neglect in transformative solutions.

Our results varied for different patterns, showing that the subtraction neglect that Adams et al.^3^ found does not generalize well on this task. Interestingly, the patterns on Levels 4 and 6, although cueing for removing tiles, did not result in any consistent tendencies to subtract for any participant group. Our interpretation is that Gestalt principles increased the additive transformations by signaling to complete a shape, but it did not provide the same cues for subtractive transformations. When pooling the data from all participant groups, we only found significant subtraction neglect effects.

### AIM3

**Figure 3:**
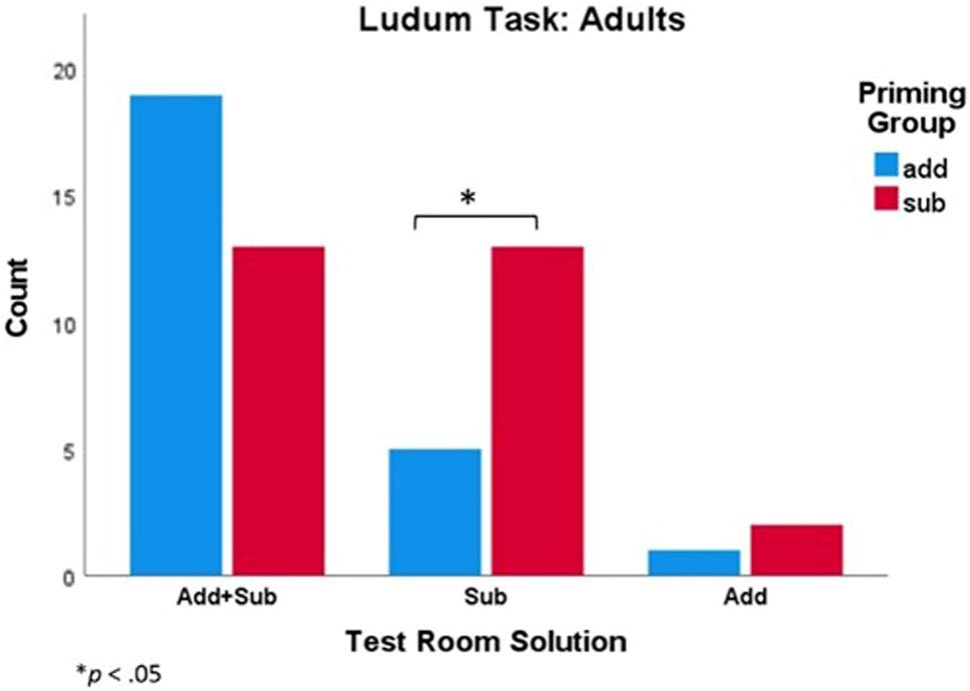
Ludum task: Addition and subtraction transformative solutions in the test room depending on the cued group for adults.

In our novel task (the Ludum game), which examined additive and subtractive cues through participants’ own generated experience rather than instruction or social learning, both subtractive or additive cued conditions influenced participants’ transformative solutions. One potential explanation for the subtractive bias that we found in the Ludum task (Fig. 3) is the role of generated own-experience and reinforcement through performance, rather than priming through instruction or through demonstration. These findings support previous work that has shown that children who utilize generated own-experience strategies in problem-solving tasks may outperform those who watch a demonstration^11,12^. In the game, before the test, participants had gone through six consecutive trials where one block was key to open the door through either an addition or subtraction transformation. It would be reasonable for individuals to assume that the next key would be the same, and thereby remove or add the block depending on their prior experience. These findings indicate that previous solutions and actions through self-experience could bias them towards similar additive or subtractive strategies. However, one limitation of the current study is that there was some given instructions (and thereby the possibility for social impact), and although we believe that the design of the task successfully minimized social effects, future studies could be designed to estimate graded responses and effect sizes of different levels of social input.

## Conclusions

Understanding how we are most likely to approach a solution to a problem has significance beyond just day-to-day living, such as in policy-making and in organizations, where human biases can cause considerable damage and resource losses. Our findings are therefore important, as they expand on previous work suggesting subtraction neglect in cognitive processes and specifically address significant concerns with the study by Adams et al.^3^.

A particularly unique contribution of the current study was the discovery that 9–10-year-old children exhibited a similar level of subtraction neglect as adults across tasks, except for the grid symmetry task, where the subtraction neglect was at a significantly higher rate than adults. This suggests that the cognitive processes associated with subtraction neglect may vary across different tasks and developmental stages. Together with previous work examining children’s problem-solving strategies^6,7^, this finding contributes to a more comprehensive understanding of how subtraction neglect manifests across development, further informing educational and cognitive research.

The findings also show nuances in how individuals approach a given task, and in many cases, a combination of both additive and subtractive solutions is chosen to maximize success. What’s more, this appears to be partly influenced by culture in the current sample that compared adults from Sweden and the United States. The social and environmental context in Sweden, such as Swedish cultural values of social equality and comprehensive and analytical approaches to problem-solving often associated with the Nordic Welfare State, may result in a cognitive approach that is less biased towards addition. However, more research is needed examining cultural factors, including social and environmental contexts, and their influence on problem-solving.

Contrary to Adams et al.^3^, we believe that a bias for subtraction neglect is not as generalizable as originally presented, but involves complex cognitive processes that are dependent on several factors. The contexts examined in this study: task dependency, culture, and age, each appear to play a role in what kind of transformative solutions are used. Despite this, we agree with Adams et al.^3^ that decisions should be based on the needs and effects of change rather than the decision-maker. We should mind the task more, not only the person.

### Supplementary Information

Supplementary Information.

## Data Availability

All data and materials are available by request (Joshua.juvrud@speldesign.uu.se). Game build file used for stimuli is available in Supplementary Materials.
