# Supplementary material for: People overlook subtractive changes differently depending on age, culture, and task
Source: Sci Rep. 2024 Jan 11;14:1086. doi: 10.1038/s41598-024-51549-y (PMC10784580; doi:10.1038/s41598-024-51549-y)
Supplement: Supplementary file 1 — Supplementary Information. [file 41598_2024_51549_MOESM1_ESM.docx]

**Method**

**Participants**

The total number of participants tested included 58 adults living in either Sweden or the United States (Swedish adults *n =* 27, American adults *n =* 31; *M* age = 21.18 years, *SD* = 1.89), and 58 children 9-10 years of age living in Sweden (*M* age = 10.10 years, *SD* = 0.21). See Table 1 in the main text for a breakdown of the sample size for each individual task.

**Lego task**

We adapted the Lego task from Adams et al. (2021) experiment 1 to additionally examine alternative explanations for performance. Participants were given a cube-shaped Lego structure with a platform on the top (Figure S1) and a cup of extra Lego bricks. The platform was supported by one small brick on the corner on top of the structure. Researchers instructed participants to stabilize the platform such that a toy car can be placed on top of it, without falling on the monkey figure standing underneath. The instructions followed a script, “You may add or take away (take away or add) Lego bricks however you like, but the structure and platform must be higher than our Lego buddy, so that they can stand underneath the platform. Try to solve the task using as few bricks as possible.” To address possible priming effects (Fischer et al., 2021), we counterbalanced the instructions as “add or take away” or “take away or add”, as well as the orientation of the Lego structure to account for possible cues for additional strategies (four counterbalance conditions).

***Figure S1.*** *The Lego structure, monkey figure, and toy car used for the Lego task.*


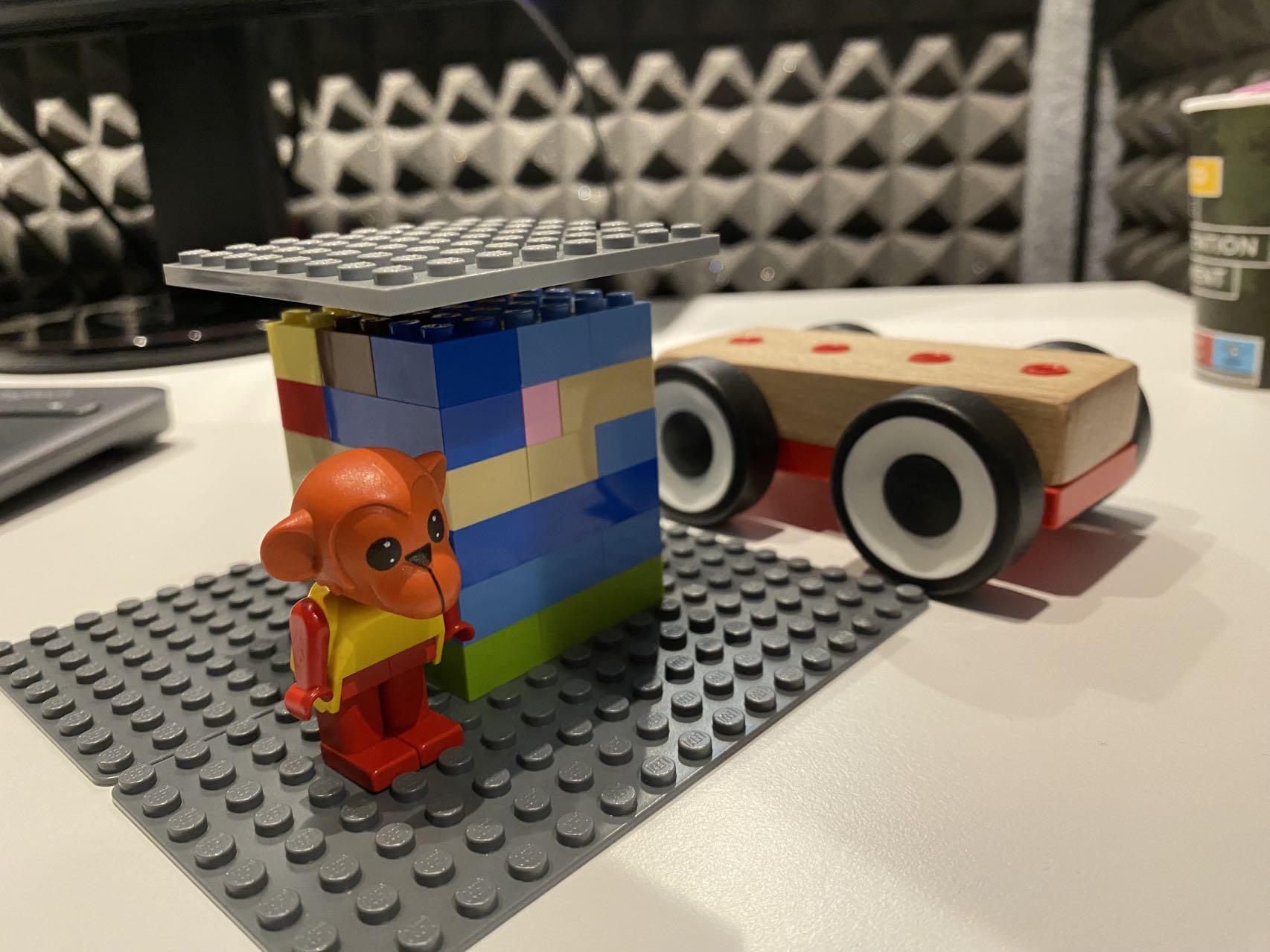


**Grid symmetry task**

We adapted the grid symmetry task from Adams et al. (2021), while also extending their findings to take into account additional configurations. In the original task, only four configurations were used, which may have limited the generalizability of the original findings., and we therefore added two more levels with gestalt laws suggesting addition, and two levels which suggested subtraction. We first provided instructions including the goal of making the quadrants symmetrical. The demonstration was counterbalanced: participants were told the task could be completed by “Tapping on an empty square to add a green tile, or by tapping a green tile to remove it” or “Tapping a green tile to remove it, or by tapping on an empty square to add a green tile.” As the researcher described adding or removing a green tile, they also demonstrated on the tablet.

Stimuli for a symmetrical pattern task were displayed on a Lenovo tablet with dimensions of 247mm x 171mm x 9.6mm. Four quadrants were shown on the screen with arrangements of green tiles. The participants were asked to make all four quadrants symmetrical about the center by tapping on empty squares to add tiles or by tapping on existing tiles to remove them. They were asked to use the fewest moves possible to complete the tasks. Once a symmetrical solution was met, the participant could move forward with the next level. The first screen acted as a practice round for the experimenter to demonstrate how the task worked. There were then eight levels for participants to work through. Patterns in each level either followed the Gestalt principles and required adding or removing tiles to yield a complete shape, or did not follow Gestalt principles. The most efficient solutions (i.e., with the fewest moves) for each level differed in the number of additive and subtractive moves (Figure S2).

***Figure S2.*** *The eight levels of the grid symmetry task, whether they followed Gestalt principles, and the number of additive and subtractive moves in their most efficient solutions. + and - represent addition and subtraction. Gestalt (+) indicates that adding would make a complete shape and Gestalt (-) indicates that subtracting would make a complete shape.*


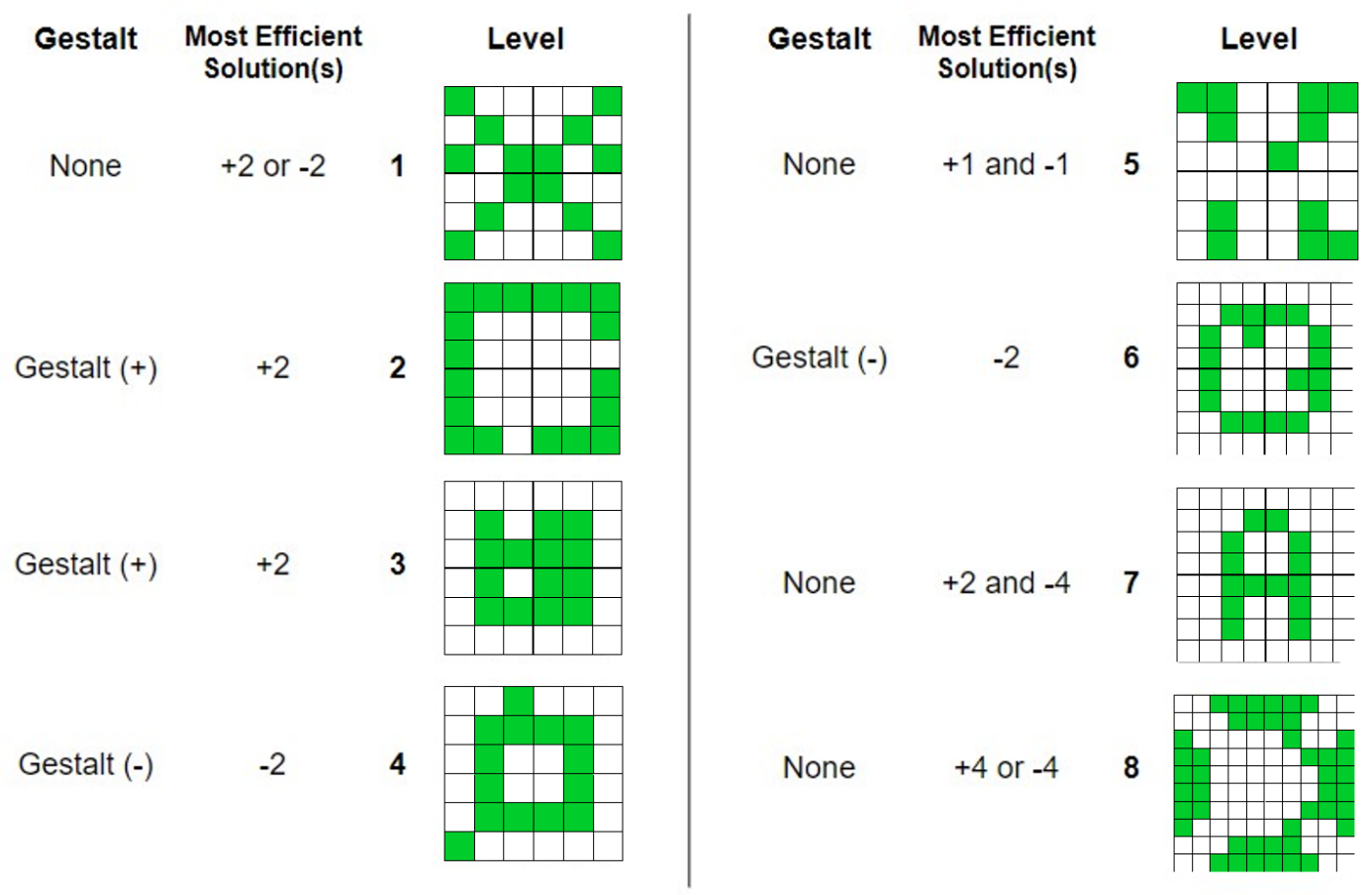


To investigate the effect of gestalt affordance we calculated a difference score between the number of additions and subtractions for each participant and level. The median was calculated separately for the of the neutral levels (L1, L5, L7 and L8) , the addition levels (L2, L3) and the subtraction levels (L4, L6), as the dependent variable in two separate repeated measures ANOVAs; one for age and one for nationality. In these analyses, only the repeated factor (gestalt) showed a significant main effect (see main text), and no main effect of age (Figure S3) or nationality (Figure S4) or their interaction with gestalt. Post-hoc analyses with Bonferroni correction showed that only addition vs subtraction differed for the age analysis (addition vs subtraction p<.001; neutral vs addition p=.087; neutral vs subtraction p=.268). For the nationality analysis, both the neutral and subtraction conditions differed from addition, but not between each other (addition vs subtraction p<.001; neutral vs addition p<.001; neutral vs subtraction p=1.000).

We also performed t-tests for the neutral levels, to stay closer to the Adams et al. study, and investigated the effect of age (only Swedes) and nationality (only adults). The t-test for age showed a significant age effect, t(68)=-2.256, p=.027, Cohen’s d=.265, but nationality was not significant, t(51)=.344, p=.732, Cohen’s d=.276. These effects can be found in figure S3 and S4 for the neutral category.

***Figure S3***. *Repeated measures for neutral / addition / subtraction levels for age, with only Swedish participants included in the analysis. Error bars denote 95% confidence intervals.*


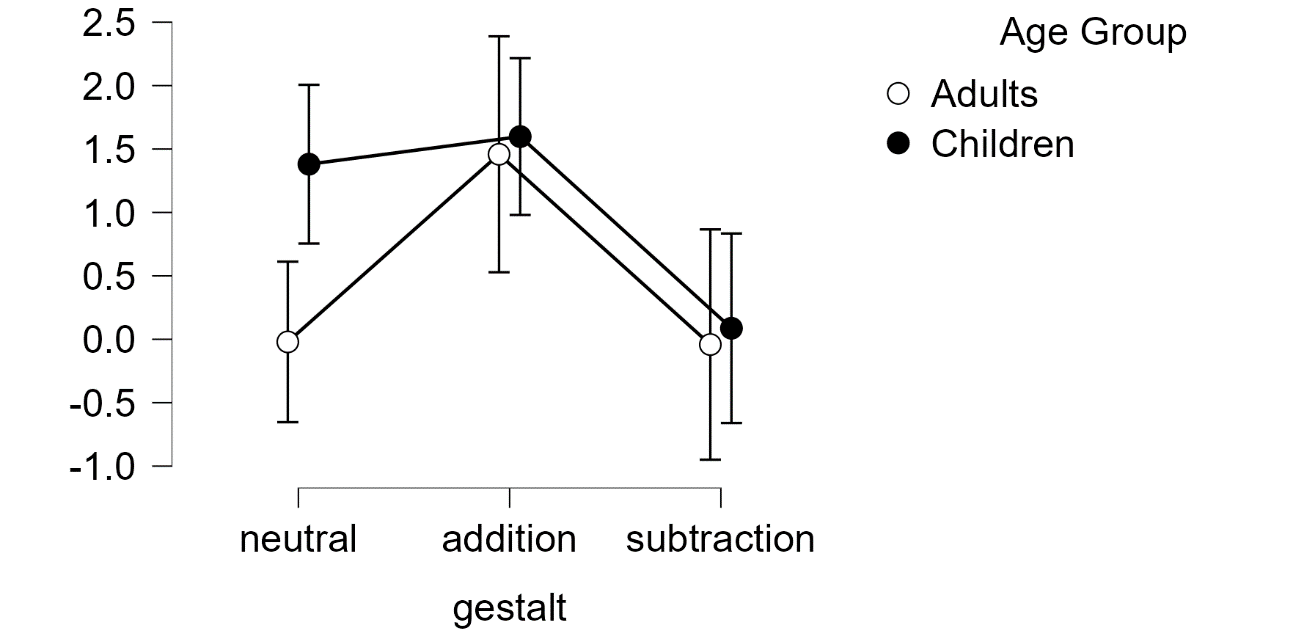


***Figure S4***. *Repeated measures for neutral / addition / subtraction levels for nationality, with only adults included in the analysis. Error bars denote 95% confidence intervals.*


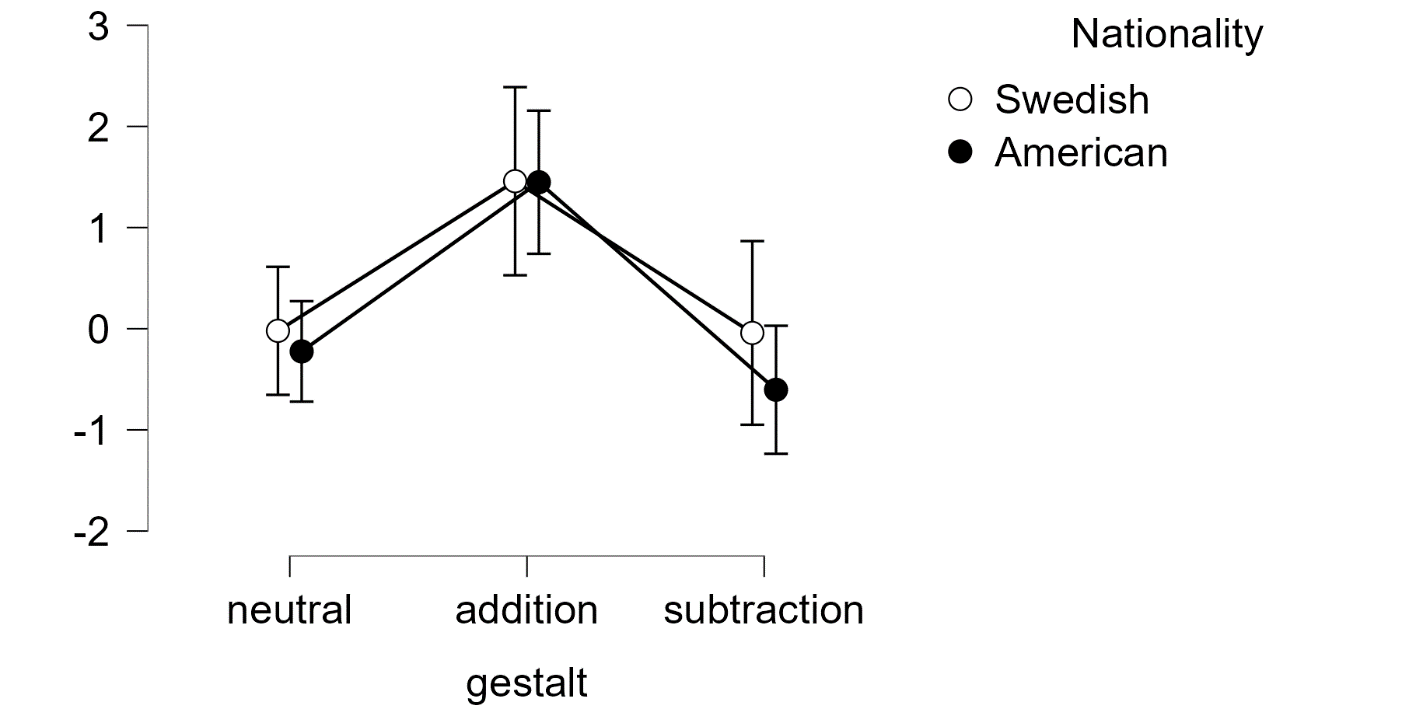


**The *Ludum* task**

Participants played a digital game called *Ludum* on a computer. The game was run using the Unity 3D game engine. The participant entered a series of rooms and interacted with objects in the rooms in order to unlock the door to the next room. They could move around using the WASD keys and interact with objects using the left mouse button.

After starting the game, researchers provided a brief description of the controls and goal of the task. Participants began the game in a practice room with no objects, and detailed written instructions of the controls were provided within the task, including how to move and orient the camera, interact with objects, and how to open and walk through the door. Researchers aided and answered questions about the controls only in the practice room.

After the practice room, participants entered a sequence of rooms with blocks of different shapes and colors, a detector platform, a red button, and a locked door. The door would only open when the participant pressed the red button while the correct combination of blocks had been placed on the detector.

There were two versions of the task: an addition cue condition and a subtraction cue condition. The two conditions differed in the training rooms and the learning phase of the task. In the training phase, participants in the addition cue condition first entered the addition rooms (Figure S5) where putting all blocks on the detector unlocks the door. In the subtractive cue condition, participants had to instead remove all blocks away from the detector to unlock the door. In the learning phase, participants had to learn which particular block opened the door. In additive rooms, a particular block had to be put on the detector, while a block had to be removed from the detector in the subtractive rooms. These rooms repeated a total of three times, with the color and shape of the blocks counterbalanced across participants and conditions.

***Figure S5.*** *Additive and subtractive cue conditions. Training phase: participants learned that adding blocks (additive condition) or subtracting blocks (subtractive condition) were required to advance to the next room. Learning phase: participants learned that adding a particular block or subtracting a particular block, but not the other block, was required to advance to the next room. This was repeated three times. Test phase: one pair of blocks were on the detector, while another pair were off the detector. Participants were required to choose a solution that either consisted of adding the learned block, or removing the learned block, or a combination of both. The test phase was identical for both the additive and subtractive conditions. Note: actual colors and shapes were randomized across participants.*

*
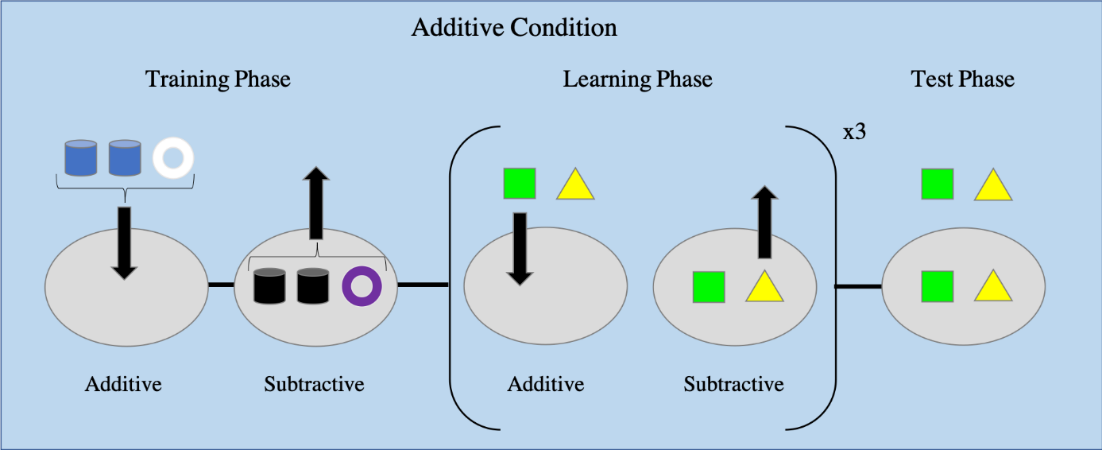

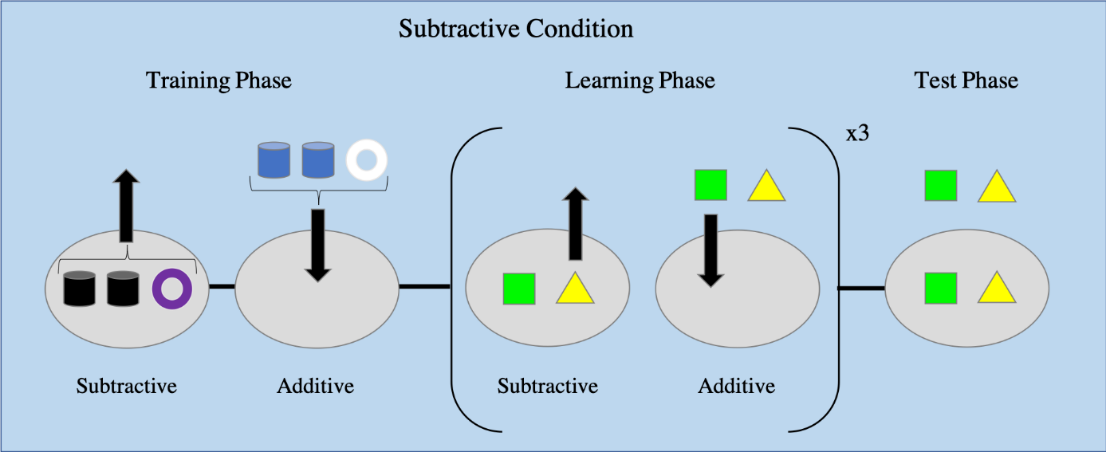
*

Finally, participants entered the test room. In the test room, there are three possible ways to open the door (see Figure 3): (1) add a block onto the detector (additive strategy), (2) remove a block from the detector (subtractive strategy), (3) both remove a block from the detector and add a block onto the detector (both subtractive and additive strategy. See Figure S6. In the test room, equal blocks were placed both on and off the detector, and thus requiring a choice of whether to add blocks, subtract blocks, or a combination of both. We determine efficiency as the number of actions required to activate the door. That is, the number of blocks added or removed. Combining both adding and subtracting blocks was therefore less efficient, because it required more actions of picking up, moving, and placing multiple blocks.

***Figure S6.*** *Possible solutions in the test room. From left to right the solutions show the subtractive strategy, the additive strategy, and both strategies.*


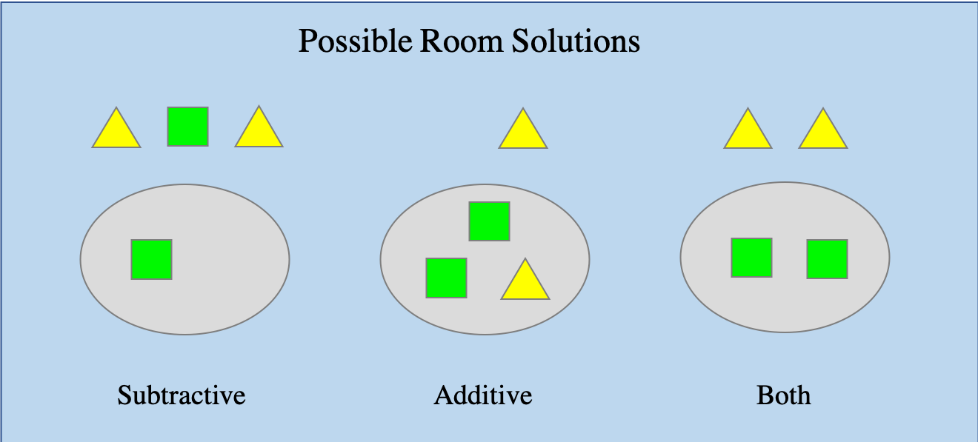


**Results and Discussion**

**Lego Task**

We first conducted a chi-squared test and found no associations between Lego strategy and the order of instructions (Χ2(2) = 4.75, *p* = .09), or the Lego strategy and the positioning of the structure (Χ2(2) = 0.31, *p* = .86). We therefore combined all data for subsequent analysis. A chi-squared test revealed no differences between the observed and expected strategy choice distribution among all of the data when coded as ‘add’, ‘sub’, and ‘both’ categories (Χ2(2) = .15, *p* = .93). This means that there was no difference between ‘add’ and ‘sub’, as well as no difference in these categories compared to a third coded category of ‘both’ (add+sub). We decided to combine the ‘add’ and ‘both’ strategy categories, as this was the less efficient strategy compared to a purely subtractive transformation and included neglect of purely subtractive solutions.. When looking at add+both compared to subtract, there was a difference between the observed and expected strategy distribution among all participants when looking at subtractions against the other two less efficient (add, both) strategies (Χ2(1) = 8.02, *p* = .005). More participants used either addition or both as their chosen strategy than was expected from chance levels (71%), and fewer used subtraction (29%). A chi-square test was used to see how this pattern continues when the participants are separated into their nationalities, exposing a significant difference (Χ2(1) = 3.68, *p* = .026). Americans accounted for most of the participants in the add-or-both category (81%), while Swedish participants accounted for most of those in the subtract category (65%).

Data for the 9-10-year-old children in the Lego task (n=22) showed that 20 children (91%) utilized an additive strategy, whereas only 2 children (9%) utilized the subtractive strategy. A chi-squared test revealed a difference between the observed and expected strategy choice distribution (Χ2(2) = 3.62, *p* < .001). An additive strategy included any final solution where extra Legos were added to the structure.

**Discussion.** Many different additive strategies were employed: adding three columns in each corner, adding one column in the corner diagonal to the preexisting column, and taking apart the base structure only to rebuild it differently and add more pieces. A subtractive strategy included any final solution where Legos were removed from the structure.

When looking at the data from all of the participants, we found no bias for addition, subtraction, or both for this task. These findings are not consistent with the ones found by Adams et al. (2021). Their design was slightly different from ours, however, because they did not have the ‘both’ category as an option. In order to make our finding more comparable to their study, we decided to look at the number of participants who used subtractions versus the participants who used a different strategy (either addition or both). After partitioning the data in this way, our results became congruent with that of Adams et al. (2021). We found that overall, there was a bias against using the subtractive transformation. When this pattern was separated by nationality, there was a difference between Americans and Swedes, in which Americans used more of the both or addition strategy and Swedes used more of the subtraction strategy. Furthermore, our design differed from Adams et al. (2021) because they also told participants that adding bricks would cost them ten cents a piece, whereas our participants had no monetary stake in transforming the structure. Adams et al. concluded that that having a subtraction cue enabled participants to recognize the subtraction solution more clearly and therefore increased the number who chose that strategy. Still, we found no difference in strategy when counterbalancing instructions. Additionally, we found that Americans use the ‘both’ strategy at a higher frequency than Swedes. Americans used either only addition or only subtraction less frequently than Swedes. These results suggest that there is a cultural difference in approaching problem solving.

**Symmetry Task**

Mean and standard deviation of the number of additive moves and subtractive moves made by participants on each level are shown in Table S1.

***Table S1.*** *Means and standard deviations for the number of additive and subtractive moves made by participants on Levels 1-8 in the grid symmetry task.*


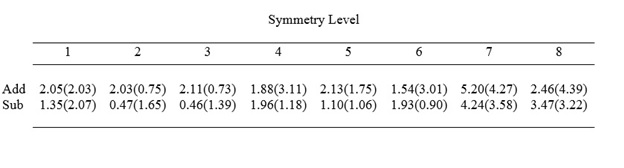


We performed paired-samples t-tests on the number of additive moves and the number of subtractive moves made by all participants (all age and culture groups included) on each level of the grid symmetry task. Participants made significantly more additive moves than subtractive moves on Level 1, t(92) = 2.24, p < .05, Cohen’s d = 0.34, Level 2, t(91) = 7.83, p < .01, Cohen’s d = 1.22, Level 3, t(91) = 10.36, p < .01, Cohen’s d = 1.49, and Level 5, t(92) = 4.70, p < .01, Cohen’s d = 0.71. Differences between the number of additive and subtractive moves on all other levels were not significant.

Paired-samples t-tests on the adult sample showed that adult participants added more than subtracted on Level 2, t(47) = 7.15, p < .01, Cohen’s d = 1.46, and Level 3, t(49) = 6.17, p < .01, Cohen’s d = 1.24. The children sample made more additive moves than subtractive moves on Level 1, t(43) = 3.61, p < .01, Cohen’s d = 0.80, Level 2, t(43) = 4.36, p < .01, Cohen’s d = 1.02, Level 3, t(41) = 9.50, p < .01, Cohen’s d = 1.89, Level 5, t(42) = 5.04, p < .01, Cohen’s d = 1.02, and Level 7, t(38) = 2.33, p < .05, Cohen’s d = 0.37.

We performed separate paired-samples t-tests on the number of additive and subtractive moves for the USA adults sample and the Swedish adults sample. Participants from both regions added more than subtracted on several levels. The USA participants made more additions than subtractions on Level 2, t(25) = 5.50, p < .01, Cohen’s d = 1.31, and Level 3, t(25) = 3.25, p < .01, Cohen’s d = 1.01. Similarly, the Swedish participants subtracted more on Level 2, t(21) = 4.50, p < .01, Cohen’s d = 1.72, and Level 3, t(23) = 7.23, p < .01, Cohen’s d = 1.57. The USA participants, however, made more subtractive moves than additive moves on Level 8, t(27) = -2.79, p = .01, Cohen’s d = 0.95, while no difference was observed for Swedish participants on the same level, t(21) = -0.11, p > .05, Cohen’s d = 0.03.

**Discussion.** Looking at all participants as a whole, there was a tendency to add more tiles than removing tiles on four of the eight levels. Among these, Level 2 and Level 3 were solved with addition-biased solutions across both nationality groups and age groups. Both levels involve patterns of an incomplete whole. These findings support the notion that the simplicity principle in Gestalt psychology would provide an explanation for the additive bias. In order to complete the shape, people were more likely to add tiles to the pattern.

The simplicity principle, however, does not fully explain our results. Levels 4 and 6 provided cues for creating a complete shape with only two tiles that needed to be removed, not added, to create the shape. If participants followed the simplicity principle on these levels, we would see a bias towards subtractive moves. This was not what we found: there was no bias for either addition or subtraction on Levels 4 and 6. The Gestalt levels that cued for addition (2 and 3) and subtraction (4 and 6) were comparable on other aspects: the number of additive/subtractive moves needed to complete the shape was the same (two), and the cued solutions were all the most efficient among all possible strategies in terms of minimizing moves. Therefore, it seems that a tendency to add more than to subtract still remained after taking into account the Gestalt principles. More specifically, additive solutions could be easily elicited by cues that rely on Gestalt principles, whereas it was harder to make people lean towards subtractive transformations with the same type of cues.

The 9-10 year old children in our sample showed a tendency to add on three more levels (1, 5, and 7) than the adults. The most efficient solutions for these levels contained either more subtractive moves (1 and 7) or the same number of additive and subtractive moves (5). It is thus reasonable to conclude that children were more biased towards additive transformations than adults, as they were prone to add even when it was not the most efficient strategy. It is also worth noting that where children had significant differences between adding and subtracting, they always favored the adding strategy,.

The American and Swedish adult samples differed only on Level 8, where the American participants were more likely to remove tiles. Removing the four outstanding tiles or adding four to create a symmetry were equally efficient for this level. This result therefore suggests that participants from the United States were slightly more likely than their Swedish counterparts to adopt subtractive strategies.

**The *Ludum* Task**

Three adult participants’ and 16 child participants’ data were excluded from the final analysis due to not reaching the test room of the task, resulting in a total adult sample of *n* = 55 and *n* = 42 children. Overall, Chi-square tests of independence revealed no difference in additive or subtractive strategies between adults and children, and no difference in additive or subtractive solutions between Swedish and American adults, X2(1) = 1.91, *p* = .17. However, when examining whether participants were primed with additive or subtractive solutions, there was a difference in the adults that were primed with subtraction solutions X2(2) = 7.29, *p* = .026, compared to additive solutions (see Table S2). We found no effect of priming for the children.

***Table S2.*** *Frequencies of addition, subtraction, or addition+subtraction solutions for the final test room in adults. Participants were primed with either addition or subtraction solutions.*

|  | | | | |
| --- | --- | --- | --- | --- |
|  | | Priming Group | | Total |
|  |  | Addition | Subtraction |  |
| Test Room Solution | Add | 2 | 3 | 3 |
|  | Sub | 5 | 13 | 19 |
|  | Add+Sub | 19 | 13 | 33 |
| Total | | 26 | 29 | 55 |
| Age group = Adult | | | | |

**Discussion.** When looking at the initial strategy for all participants, these results do not support the findings from Adams et al. (2021): participants were more likely to subtract rather than add. This finding did not vary across nationality, suggesting that culture did not affect additive and subtractive choices. No difference appeared between conditions, showing that an initial cue did not affect participants’ first choice to add or subtract. We found a marginal difference, however, between the condition and final solution. Those in the addition condition went through a room they needed to subtract in directly before the test room, and the opposite happened for those in the subtraction condition. Although this effect was marginal, it suggests that the cue directly before the test room might be more important in determining someone’s strategy than the initial example.

**Materials (The *Ludum* task) S1.** To download a demonstration of The *Ludum* task, visit: <https://drive.google.com/file/d/10X9juoQBqjvDPxdXMgxNkCADNRQNe-SR/view?usp=sharing>

For video demonstrations of the two conditions, visit: https://drive.google.com/drive/folders/1malJZAAqTwrGbNW_5DagUJI4EJOL9TIa?usp=drive_link
